# Supplementary material for: A Systematic Review of Oral Vertical Dyskinesia (“Rabbit” Syndrome)
Source: Medicina (Kaunas). 2024 Aug 19;60(8):1347. doi: 10.3390/medicina60081347 (PMC11355986; doi:10.3390/medicina60081347)
Supplement: Supplementary file 1 [file medicina-60-01347-s001.zip › medicina-3146642-supplementary.pdf]

Supplementary Material

Table S1. FreeText and MeSH search terms in the U.S. National Library of Medicine.
Table S2. Literature review of the cases of RS.

Table S1. FreeText and MeSH search terms in the U.S. National Library of Medicine.

Query	Mesh terms	Results
"rabbit syndrome"	("rabbit s"[All Fields] OR "rabbits"[MeSH Terms] OR "rabbits"[All Fields] OR "rabbit"[All Fields]) AND ("syndrom"[All Fields] OR "syndromal"[All Fields] OR "syndromally"[All Fields] OR "syndrome"[MeSH Terms] OR "syndrome"[All Fields] OR "syndromes"[All Fields] OR "syndrome s"[All Fields] OR "syndromic"[All Fields] OR "syndroms"[All Fields])	6334

Table S2. Literature review of the cases of RS.

Reference	Age/ Sex	Diagnosis	Cause	DUR[I]	Management	OUT	PD[II]	TD[III]
Villeneuve *et al*. (1972) [1]	66/ F	SCZ	PPZ	NA	PPZ dose reduced. THP and BZT were STT.	FR	Y	N
	67/ F	SCZ	TFP	NA	BZT was STT.	FR	Y	Y
	54/ F	SCZ	CLP, TFP, THZ	NA	PCD was STT.	PR	Y	N
	66/ M	SCZ	TFP, LVP, BZT	NA	THP was STT.	PR	N	Y
	47/ M	SCZ	THZ	NA	PCD was STT.	PR	Y	N
Jus *et al*. (1974) [23]	NA/ M	NA	NA	NA	NA	NA	N	N
	NA/ M	NA	NA	NA	NA	NA	N	N
	NA/ M	NA	NA	NA	NA	NA	N	N
	NA/ F	NA	NA	NA	NA	NA	N	N
	NA/ F	NA	NA	NA	NA	NA	N	N
	NA/ F	NA	NA	NA	NA	NA	N	N
Sovner *et al*. (1977) [24]	54/ F	SCZ	FLP	8 mo	BZT was STT.	FR	N	Y
Weiss *et al*. (1980) [25]	62/ F	SCZ	Mesoridazine	18 mo	PST improved symptoms. THP was not effective.	PR	Y	Y
	68/ F	SCZ	THZ	10 yr	PST worsened symptoms. THP was effective.	PR	Y	Y
Gangadhar *et al*. (1981) [26]	38/ M	Acute psychotic disorder	HAL, FLP	6 d	HAL and FLP were d/c. THP was STT.	FR	Y	N
Kachi *et al*. (1981) [27]	NA	NA	HAL	NA	NA	NA	Y	N
Kakigi *et al*. (1982) [28]	NA	NA	SUL	NA	NA	NA	NA	NA

Todd *et al*. (1983) [29]	63/ F	SCZ	FLP	8 d	BZT was STT.	PR	Y	N
	66/ M	BPD	HAL	8 d	HAL was SWT to CLP. BZT was STT. Amantadine and BZD were not effective.	PR	Y	N
Yassa *et al*. (1986) [9]	42/ F	SCZ	HAL	16 mo	PCD was STT.	FR	Y	N
	50/ F	SCZ	HAL	9 mo	PCD was STT.	FR	N	N
	57/ M	SCZ	HAL	7 mo	PCD was STT.	FR	Y	N
	67/ F	BPD	HAL	8 mo	PCD was STT.	FR	Y	N
	71/ F	Korsakoff's syndrome	HAL, CLP	12 mo	PCD was STT.	FR	Y	N
	77/ M	Cognitive impairment	HAL	18 mo	PCD was STT.	FR	Y	N
Decina *et al*. (1990) [12]	59/ F	SCZ	HAL	NA	NA	NA	Y	N
	55/ M	SCZ	HAL	NA	NA	NA	Y	N
	NA	SCZ	NA	NA	NA	NA	N	Y
Deshmukh *et al*. (1990) [30]	52/ M	SCZ	TFP	NA	TFP was d/c. THP was STT.	FR	N	N
Truong *et al*. (1990) [31]	74/ F	NA	IDP	NA	NA	NA	NA	NA
Almeida *et al*. (1991) [32]	75/ M	SCZ	TFP, FLP	Mo	PCD was STT.	FR	N	N
Fornazzari *et al*. (1991) [15]	45/ F	MDD	IMP	4 yr	IMP SWT by phenelzine.	FR	N	N
Inada *et al*. (1991) [11]	55 (mean)/ 9 M and 8 F	13 SCZ, 2 cognitive impairment, and 2 BPD	NA	NA	NA	NA	14Y	NA
Wada *et al*. (1992) [16]	63/ F	SCZ	HAL, LVP, lithium	11 yr	THP was STT.	PR	Y	N
	63/ F	SCZ	Propericiazine, clocapramine	32 yr	THP was STT.	FR	N	Y

	44/ F	SCZ	HAL, propericiazine, SUL	13 yr	BIP was STT.	FR	Y	N
	48/ F	SCZ	HAL, LVP, SUL	31 yr	BIP was STT.	PR	Y	N
	58/ M	SCZ	Bromperidol, SUL	11 yr	THP was STT.	FR	Y	N
Chiu *et al*. (1993) [10]	77/ F	SCZ	THZ	15 mo	THP was STT.	FR	N	N
Kelvin *et al*. (1993) [33]	76/ F	MDD	Lofepramine	NA	PCD was STT.	No	N	N
Nishiyama *et al*. (1993) [34]	61/ F	NA	IDP	NA	NA	NA	NA	NA
Goswami *et al*. (1994) [35]	NA	NA	NA	NA	NA	NA	NA	NA
Poungvarin *et al*. (1994) [36]	50/ M	NA	IDP	NA	BZT was STT.	FR	N	N
Schwartz *et al*. (1995) [37]	56/ F	Acute paranoid episode	HAL	NA	NA	NA	N	N
Gada *et al*. (1997) [38]	46/ F	SCZ	Flupenthixol	1 yr	Flupenthixol was d/c. THP was STT.	FR	N	N
Hayashi *et al*. (1997) - Case 2 [39]	53/ F	SCZ	Oxypertine, clonazepam	7 mo	Oxypertine was d/c. THP was STT.	PR	N	Y
Schwartz *et al*. (1998) [40]	68/ F	SCZ	RIS	4 mo	BZX was STT.	FR	Y	N
Kamijo *et al*. (1999) [41]	65/ F	Suicide attempt	Phenol ingestion	NA	NA	NA	Y	N
Levin *et al*. (1999) [42]	27/ M	SCZ	RIS	7 mo	RIS dose was decreased. BIP was STT.	PR	N	N
Miwa *et al*. (1999) [43]	36/ F	NA	IDP	NA	Tiapride was STT.	FR	N	N
Choi et al. (2000) [44]	28/ M	Aggressive behavior	NA	NA	BZT was STT.	FR	Y	N

Damodaran *et al*. (2000) [45]	31/ F	SCZ	HAL	Yr	HAL SWT to OLZ.	FR	N	N
Durst *et al*. (2000) [46]	28/ F	SCZ	Zuclopenthixol, BIP	Yr	Zuclopenthixol was SWT by OLZ.	FR	N	N
Nishimura *et al*. (2001) [47]	38/ F	SCZ	RIS	2 mo	RIS was d/c. THP was STT.	FR	N	Y
Sethi *et al*. (2001) [48]	24/ M	BPD	HAL	1 wk	THP was STT.	FR	Y	N
Hoy *et al*. (2002) [49]	38/ M	MDD with psychotic features	RIS	4 mo	BZT was STT.	FR	N	N
Ortín Castaño *et al*. (2003) [50]	68/ F	Gastrointestinal disorder	Clebopride	1 yr	Clebopride was d/c.	FR	Y	N
Sethi *et al*. (2003) [51]	48/ F	SCZ	CLZ	7 mo	THP was STT.	FR	N	Y
Eren *et al*. (2004) [52]	56/ M	MDD with psychotic features	RIS	4 mo	RIS was d/c.	FR	Y	N
Gourzis *et al*. (2004) [53]	65/ F	MDD	PPZ, AMT, and paroxetine	1 mo	Paroxetine was d/c.	FR	N	N
Mendhekar *et al*. (2004) [54]	29/ F	Schizophreniform disorder	ARIP	6 wk	ARIP dose was reduced. THP was STT.	FR	Y	N
Altindag *et al*. (2005) [55]	22/ M	SCZ	RIS, BIP	12 wk	RIS was SWT by QTP.	PR	Y	N
Mendhekar *et al*. (2005) [56]	42/ F	BPD	RIS	2 wk	RIS was d/c. PMT was STT.	FR	N	Y
Sabolek *et al*. (2005) [57]	74/ F	BPD	OLZ	NA	NA	NA	NA	NA
Parvin *et al*. (2005) [58]	41/ F	MDD	ESC	2 wk	ESC was d/c. BZD was STT.	FR	N	N
	24/ M	OCD	Citalopram	10 d	Citalopram was d/c.	FR	N	N
Mendhekar *et al*. (2006) [59]	6/ M	Attention-deficit/ hyperactivity disorder	Methylphenidate	7 d	Methylphenidate was d/c.	FR	N	N

Zacher *et al.* (2006) [60]	27/ F	BPD	ARIP	2 mo	ARIP was SWT to ziprasidone. BZT was STT.	FR	Y	N
Catena Dell'osso *et al.* (2007) [2]	50/F	BPD	RIS	3 mo	NA	NA	Y	N
Gonidakis *et al.* (2008) [61]	35/ F	SCZ	ARIP	3 mo	ARIP dose was reduced. BIP was STT.	FR	N	N
Praharaj *et al.* (2008) [62]	35/ M	Schizoaffective disorder	OLZ	2 wk	OLZ dose was reduced.	PR	Y	N
Wu *et al.* (2008) [63]	56/ F	BPD	QTP	2 wk	QTP was SWT by OLZ.	FR	N	N
Hocaoglu *et al.* (2009) [64]	44/ F	SCZ	CLZ	10 mo	CLZ was SWT to QTP.	PR	N	N
Caykoylu *et al.* (2010) [65]	45/ F	Unspecified psychotic disorder	ARIP	3 mo	ARIP was d/c. BIP was STT.	FR	Y	N
Mendhekar *et al.* (2010) [66]	78/ M	MDD	AMI	5 d	AMI was d/c. THP was STT.	FR	Y	N
Cumurcu *et al.* (2011) [67]	56/ F	MDD	QTP	2 wk	QTP was d/c. BZD was STT.	FR	N	N
Teng *et al.* (2011) [68]	64/ F	SCZ	PLP	1 yr	PLP was SWT by OLZ.	FR	N	N
Gray *et al.* (2012) [69]	58/ F	MDD	Sertraline	2 d	Sertraline was d/c. BZT was STT.	FR	Y	N
Kunjithapatham *et al.* (2012) [70]	32/ F	BPD	OLZ	NA	OLZ dose reduced. BZX was STT.	FR	N	N
Lay *et al.* (2012) [71]	28/ F	OCD	RIS	NA	RIS was SWT by QTP.	FR	N	N
Liang *et al.* (2012) [21]	56/ M	BPD	PLP	NA	BIP was STT.	FR	N	N
Sansare *et al.* (2012) [72]	54/ F	NA	RIS	NA	NA	NA	NA	NA
	NA	NA	RIS	NA	NA	NA	NA	NA

Garg *et al*. (2013) [20]	22/ F	BPD	Levosulpiride	2 mo	Levosulpiride was d/c. BZX was STT.	FR	N	N
Callista *et al*. (2014) [73]	29/ F	Headache	AMT	1 mo	AMT was d/c. BIP was STT.	FR	N	N
Nimber *et al*. (2014) [74]	35/ M	MDD	ESC	3 mo	ESC was d/c. THP was STT.	FR	N	N
Dey *et al*. (2015) [75]	16/ M	Inhalant use disorders	ARIP	7 d	ARIP was d/c. BZD and THP were STT.	FR	N	N
Kitamoto *et al*. (2015) [76]	48/ F	Suicide attempt	2-(4-chloro2-methylphenoxy propionic) acid	NA	NA	NA	NA	NA
Nagar *et al*. (2015) [77]	25/ M	Hair loss	MXF	1 wk	MXF was d/c.	FR	N	N
	32/ M	Hair loss	MXF	1 d	MXF was d/c.	FR	N	N
Nataraj *et al*. (2015) [78]	11/ F	BPD	RIS	4 mo	RIS was d/c.	FR	N	N
Park *et al*. (2016) [79]	23/ M	NA	Right vertebral artery compression	NA	NA	NA	NA	NA
Belli *et al*. (2017) [80]	58/ F	BPD	QTP	12 wk	QTP was SWT to OLZ.	FR	N	N
Reichenberg *et al*. (2017) [81]	31/ F	MDD	Lurasidone	6 mo	Lurasidone SWT by OLZ.	FR	Y	Y
Soyer *et al*. (2017) [82]	63/ M	Chronic hepatitis C	IFN	1 wk	IFN was d/c.	FR	N	N
Kuo *et al*. (2018) [83]	90/ F	NA	IDP	NA	Levodopa was STT.	FR		
Rebello *et al*. (2018) [19]	47/ F	BPD	RIS	Yr	RIS was d/c. THP was STT.	FR	N	Y
Albayrak *et al*. (2019) [84]	62/ M	Brief psychotic disorder	RIS	1 mo	RIS was d/c. BIP and BZD failure. Fluvoxamine was STT.	FR	N	N
Demiröz *et al*. (2019) [85]	40/ M	BPD	ARIP	4 wk	ARIP was d/c. BIP and BZD were STT.	FR	N	N

Erguner *et al*. (2019) [86]	46/ F	OCD	AMI	18 d	AMI was d/c.	FR	N	N
Datta *et al*. (2020) [87]	16/ F	NA	NMDA	NA	NA	NA	NA	NA
Roy *et al*. (2020) [88]	56/ F	BPD	OLZ	1 yr	OLZ was d/c. PMT and tetrabenazine were STT.	FR	Y	N
Aniello *et al*. (2021) [89]	65/ F	NA	Functional	NA	NA	NA	NA	NA
dos Santos Souza *et al*. (2021) [90]	89/ F	Behavioral changes and aggression	RIS	4 mo	RIS was SWT by OLZ.	FR	Y	N
Sastry *et al*. (2021) [91]	24/ F	Headache	ESC	1 d	ESC was d/c. THP was STT.	FR	N	N
Gundogmus et al. (2022) [92]	28/ F	SCZ	AMI	6 wk	AMI was SWT to OLZ.	FR	N	N
Naguy *et al*. (2022) [93]	11/ M	OCD	FLX	2 wk	FLX dose decreased.	FR	N	N
Batra *et al*. (2023) [94]	65/ F	Insomnia	ESC	3 d	ESC was d/c.	FR	N	N
Hsieh *et al*. (2024) [95]	53/ M	BPD	ARIP	7 mo	NA	NA	NA	NA
Huynh *et al*. (2024) [96]	45/ F	SCZ	HAL	10 d	NA	NA	NA	NA

Abbreviations: AMI, amisulpride; AMT, amitriptyline; ARIP, aripiprazole; BPD, bipolar disorder; BIP, biperiden; BZD, benzodiazepine; BZT, benztropine; BZX, benzhexol; CLZ, clozapine; CLP, chlorpromazine; d, day(s); d/c, discontinued; DUR, duration; EEG, electroencephalogram; ESC, escitalopram; F, female; FLP, fluphenazine; FLX, fluoxetine; FR, full recovery; HAL, haloperidol; IDP, idiopathic; IFN, peginterferon-α; IMP, imipramine; LVP, levomepromazine; M, male; mo, month(s); MDD, major depressive disorder; MXF, minoxidil foam; N, no; NA, not available/ not reported; NMDA, anti-N-methyl-D-aspartate receptor encephalitis; OCD, obsessive-compulsive disorder; OLZ, olanzapine; OUT, outcome; PCD, procyclidine; PD, Parkinson's disease; PLP, paliperidone; PMT, promethazine; PPZ, perphenazine; PR, partial recovery; PST, physostigmine; QTP, quetiapine; RIS, risperidone; SCZ, schizophrenia; STT, started; SUL, sulpiride; SWT, switched; TD, tardive dyskinesia; TFP, trifluoperazine; THP, trihexyphenidyl; THZ, thioridazine; wk, week(s); Y, yes; yr, year(s).

[I] Duration of therapy

[II] Parkinson's disease symptoms, including resting tremor, bradykinesia, and rigidity

[III] Tardive dyskinesia symptoms, including dyskinetic movements in extremities and tongue
